# Does surgical indication influence implant survival in reverse shoulder arthroplasty? A comparative cohort study of fracture and glenohumeral osteoarthritis

**DOI:** 10.1007/s00590-026-04815-w

**Published:** 2026-06-04

**Authors:** Luboš Nachtnebl, Vasileios Apostolopoulos, Pavel Brančík, Radka Čechová, Martin Dajča, Vladimír Červeňák, Lukáš Martinek, Michal Mahdal, Tomáš Tomáš

**Affiliations:** 1 First Department of Orthopaedic Surgery, St. Anne´s University Hospital, Brno, Czech Republic; 2 Department of Radiology, St. Anne´s University Hospital, Brno, Czech Republic

**Keywords:** Reverse shoulder arthroplasty, Proximal humeral fracture, Glenohumeral osteoarthritis

## Abstract

**Background:**

Reverse shoulder arthroplasty (RSA) is widely used for the treatment of advanced glenohumeral osteoarthritis and complex proximal humeral fractures. However, comparative data evaluating implant survival and outcomes between fracture and elective indications remain limited. This study aimed to assess mid- to long-term implant survival, complications, radiographic findings, and functional outcomes following RSA, with particular attention to the influence of surgical indication.

**Methods:**

A retrospective cohort study included 126 patients who underwent primary RSA using a single implant system. Patients were treated for either advanced glenohumeral osteoarthritis or complex proximal humeral fractures. Implant survival was analyzed using Kaplan–Meier estimates, and potential predictors were evaluated with Cox regression analysis. Secondary outcomes included complications, radiographic changes, Oxford Shoulder Score (OSS), and range of motion (ROM). The mean follow-up was 57.4 ± 51.1 months.

**Results:**

The overall one-year implant survival rate was 96.8%, declining to 93.4% at eight years and remaining stable thereafter. Survival differed numerically by diagnosis, reaching 98.3% for osteoarthritis and 86.4% for fractures at eight years. No statistically significant predictors of failure were identified; however, uncemented stems showed a numerically higher risk of events (HR 3.01, p = 0.088), and fracture indication demonstrated a numerically higher risk compared with osteoarthritis (HR 2.10, p = 0.220). The overall complication rate was 7.14%. The mean OSS was 46.34 ± 4.72. Postoperative ROM values were within expected ranges, with no significant differences between groups.

**Conclusion:**

Reverse shoulder arthroplasty demonstrated satisfactory mid- to long-term implant survival and favorable postoperative outcomes in both fracture and elective indications. Although fracture cases showed numerically lower survivorship, no statistically significant independent effect of surgical indication on implant failure was demonstrated in this cohort. The findings should be interpreted with caution given the retrospective design, limited number of revision events, and absence of preoperative functional data.

## Introduction

Reverse shoulder arthroplasty represents a well-established surgical modality with a fundamental role in contemporary orthopedic surgery. Although reverse shoulder arthroplasty was originally designed for the treatment of cuff-deficient glenohumeral osteoarthritis, the indications have considerably expanded [1]. Numerous studies have found superior outcomes for proximal humeral fractures treated with RSA compared with other surgical interventions [2]. However, there are a few studies comparing outcomes of RSA for proximal humeral fractures with RSA for elective indications [3–6]. Recent registry-based and large-cohort studies have further evaluated the impact of humeral stem fixation in reverse shoulder arthroplasty [2, 3, 7, 8].

Outcomes after reverse shoulder arthroplasty are influenced by multiple factors related to patient characteristics, surgical technique, and implant design [9, 10]. Implant-related variables, including component design, size, and fixation method, may affect postoperative stability, complication rates, and long-term implant survival [11–13]. Studies have suggested that, compared with standard humeral stems, short humeral stems, particularly those implanted using a press-fit technique, are safe and may be associated with lower complication rates [7, 8, 14]. Furthermore, procedure-specific complications such as scapular notching continue to be reported and may have a substantial impact on functional outcomes and patient satisfaction [15, 16]. Previous studies on reverse shoulder arthroplasty have often involved heterogeneous populations, varying implant designs, or focused on isolated outcomes, with limited direct comparison between fracture and elective indications under standardized conditions [11, 12]. This study provides a comprehensive evaluation of implant survival, complications, radiographic findings, and functional outcomes within a single-cohort setting using a uniform implant system and surgical technique.

Given the expanding indications for reverse shoulder arthroplasty and the ongoing debate regarding outcomes in fracture versus elective cases, further comparative data are warranted. The primary research question of this study was whether surgical indication (complex proximal humeral fracture versus glenohumeral osteoarthritis) influences implant survival following reverse shoulder arthroplasty. We hypothesized that implant survival would be comparable between indications when using a standardized surgical technique and a single implant system. Secondary objectives included the evaluation of complications, radiographic findings, and functional outcomes, as well as the potential influence of implant-related variables such as fixation method and stem design.

## Methods

We performed a retrospective cohort study of consecutive patients treated with primary RSA Affinis (Enovis) implants at a single tertiary orthopedic center. Patients were divided into two groups according to the primary indication: (1) advanced glenohumeral osteoarthritis (elective RSA) and (2) complex proximal humeral fracture (fracture RSA).

A total of 126 patients who underwent RSA for either advanced glenohumeral osteoarthritis or complex proximal humeral fracture were included. Eligibility criteria were: primary RSA for one of the two predefined indications and availability of postoperative follow-up data (clinical and/or radiographic). Exclusion criteria included revision RSA at index surgery, extensive proximal humerus fractures that required revision systems, acute infection, tumor surgery, and missing essential follow-up information for survival/complication analyses (Table 1).

**Table 1:** Baseline characteristics of the dataset stratified by surgical indication

	Overall	Glenohumeral osteoarthritis	Fracture	*p* values
Number of patients, n (%)	126 (100%)	65 (51.6%)	61 (48.4%)	
Age, years (mean ± SD)	69.6 ± 10.2	68.5 ± 8.5	70.7 ± 11.6	0.11
Gender, n (%)				0.44
Male	35 (27.8%)	20 (30.8%)	15 (24.6%)	
Female	91 (72.2%)	45 (69.2%)	46 (75.4%)	
Glenosphere diameter, mm (mean ± SD)	40.0 ± 1.7	40.0 ± 1.8	40.0 ± 1.5	0.81
Stem size, mm (mean ± SD)	10.9 ± 2.3	11.2 ± 2.3	10.7 ± 2.3	0.23
Offset / Inlay, n (%)				0.32
0 mm	28 (22.2%)	17 (26.2%)	11 (18.0%)	
3 mm	43 (34.1%)	23 (35.4%)	20 (32.8%)	
6 mm	43 (34.1%)	20 (30.8%)	23 (37.7%)	
9 mm	9 (7.1%)	5 (7.7%)	4 (6.6%)	
12 mm	3 (2.4%)	0 (0.0%)	3 (4.9%)	
Stem fixation, n (%)				< 0.001
Cemented	95 (75.4%)	37 (56.9%)	58 (95.1%)	
Uncemented	31 (24.6%)	28 (43.1%)	3 (4.9%)	
Follow-up, months (mean ± SD)	57.4 ± 51.1	60.5 ± 55	50.4 ± 46	0.44

### Evaluation

All procedures were performed by experienced shoulder surgeons using a standardized deltopectoral approach. Implant-related variables recorded for analysis included humeral stem fixation method (cemented vs uncemented), humeral stem size/length, and glenosphere diameter. Perioperative complications were documented from operative notes and inpatient records. The choice of humeral stem fixation (cemented vs uncemented) was determined intraoperatively by the operating surgeon based on bone quality, patient characteristics, and surgical indication.

Patients were followed clinically and radiographically at routine postoperative intervals. The primary endpoint was implant survival, defined as freedom from revision surgery or implant removal for any reason. Secondary endpoints included complications (intraoperative and postoperative), radiographic changes (scapular notching and radiolucent lines), and functional outcomes. The minimum postoperative follow-up was 12 months.

Radiographic evaluations were performed by a single experienced investigator, blinded to clinical outcomes, using standardized imaging protocols. All radiographs were obtained according to standardized protocols (true AP projection and axial), which allowed us to evaluate two key parameters—scapular notching and radiolucent lines [17]. During the imaging, patients stood in an upright position with a slight rotation towards the prosthesis with the arm in slight abduction. The images were performed during exhalation to eliminate the possibility of summation of the prosthesis with the scapula or irrelevant overlapping anatomical structures. We evaluated scapular notching according to the Nerot-Sirveaux classification [18]. This uses the inferior screw of the scapular portion of the prosthesis as the central point. In our dataset, the inferior pin was considered equivalent to the inferior screw for classification purposes.

Functional outcomes were assessed using the Oxford Shoulder Score (OSS) at the latest available follow-up. Functional outcome analyses were performed using complete-case data. Missing data were not imputed due to the retrospective design and the nature of clinical and patient-reported outcomes. Active range of motion (ROM) was recorded in degrees for flexion, extension, abduction, adduction, external rotation, and internal rotation using standardized clinical measurement (goniometer-based assessment) by trained clinicians. Direct comparison of preoperative functional status between fracture and elective indications would not be methodologically appropriate due to the fundamentally different baseline clinical conditions. Complications were defined as any intraoperative or postoperative adverse events related to the procedure or implant and were subsequently classified by timing and type. Infectious complications were defined based on clinical signs, laboratory parameters (including inflammatory markers), and/or the need for surgical revision, as documented in medical records. All suspected cases were evaluated according to standard institutional protocols.

### Statistical analysis

Implant survival was estimated using Kaplan–Meier analysis with time-to-event defined from index RSA to revision/extraction or last follow-up (censored). Potential predictors of failure were evaluated using Cox proportional hazards regression, including fixation type (cemented vs uncemented), diagnosis (fracture vs osteoarthritis), stem size/length, glenosphere size, age, and sex. Results were reported as hazard ratios (HR) with 95% confidence intervals (CI). Continuous variables (OSS, ROM) were summarized as mean ± standard deviation; categorical variables (complications, notching grades) were summarized as counts and percentages. A two-sided *p* value < 0.05 was considered statistically significant. Analyses were performed using standard statistical software R (RStudio).

## Results

### Implant survival

**Fig. 1:**
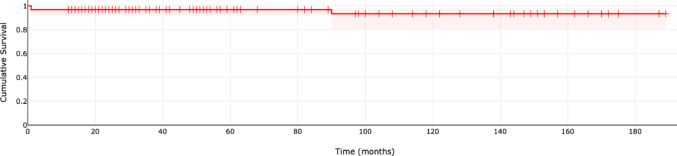
Cumulative implant survival of the RSA

A total of 6 revision events (implant failures) were observed during follow-up. The one-year implant survival rate was 96.8% (number at risk: 105). At eight years, the survival rate declined to 93.4% (number at risk: 43) and remained stable at this level through the follow-up period (Fig. 1).

**Fig. 2:**
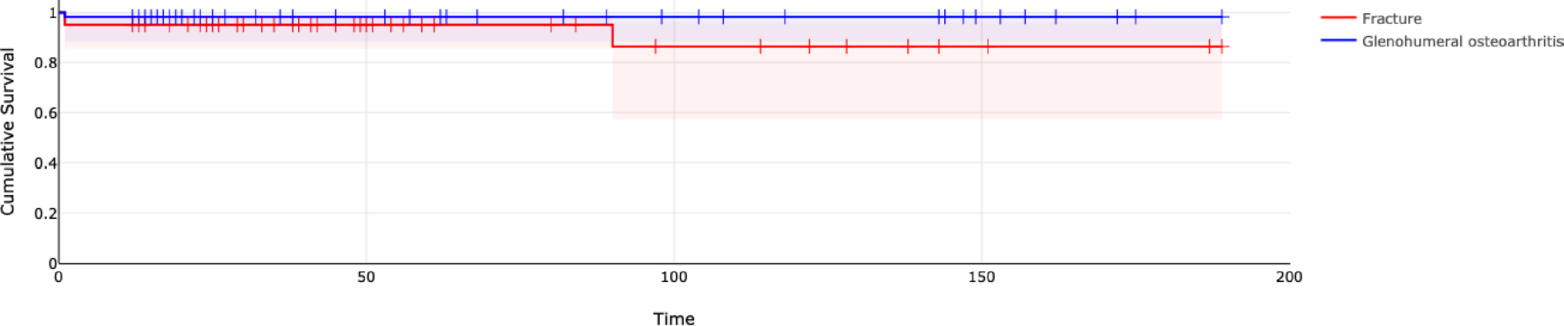
Comparison of cumulative implant survival: glenohumeral osteoarthritis vs. fracture

Implant survival rates differed numerically by diagnosis. While glenohumeral osteoarthritis maintained a consistent survival rate of 98.3% throughout follow-up (number at risk: 60 at one year; 25 at eight years), fracture-related implants showed a greater decline. Specifically, survival in the fracture group decreased from 95.1% at one year (number at risk: 55) to 86.4% at eight years (number at risk: 18), remaining stable thereafter (Fig. 2).

**Fig. 3:**
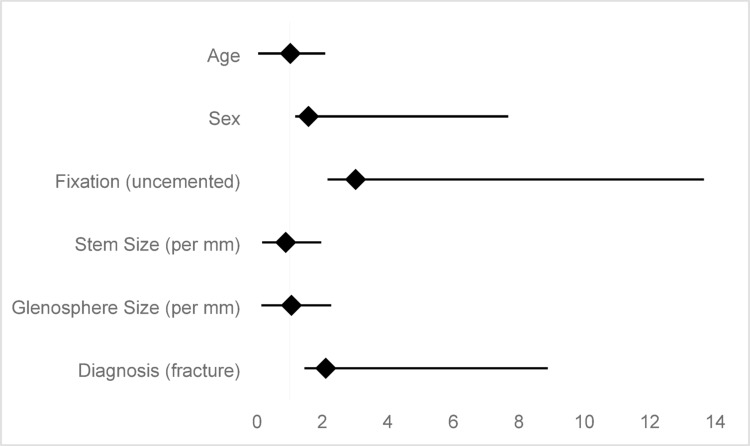
Association between risk factors and implant survival in RSA

Among the factors examined, no statistically significant predictors of implant failure were identified. Uncemented fixation demonstrated a higher risk of events compared with cemented stems (HR 3.01, 95% CI 0.85–10.64; p = 0.088). Similarly, fracture indication was associated with an increased risk compared with glenohumeral osteoarthritis (HR 2.10, 95% CI 0.65–6.78; p = 0.220). Increasing stem size showed a trend toward reduced risk (HR 0.88, 95% CI 0.72–1.08; p = 0.218), while glenosphere size had no apparent effect on implant survival (HR 1.05, 95% CI 0.91–1.21; p = 0.512). Neither age (HR 1.02, 95% CI 0.98–1.06; p = 0.352) nor sex (HR 1.57, 95% CI 0.40–6.10; p = 0.521) were associated with implant failure. Although not statistically significant, the observed trends suggest a potential influence of fixation type and surgical indication on implant survival (Fig. 3).

### Complications

**Fig. 4:**
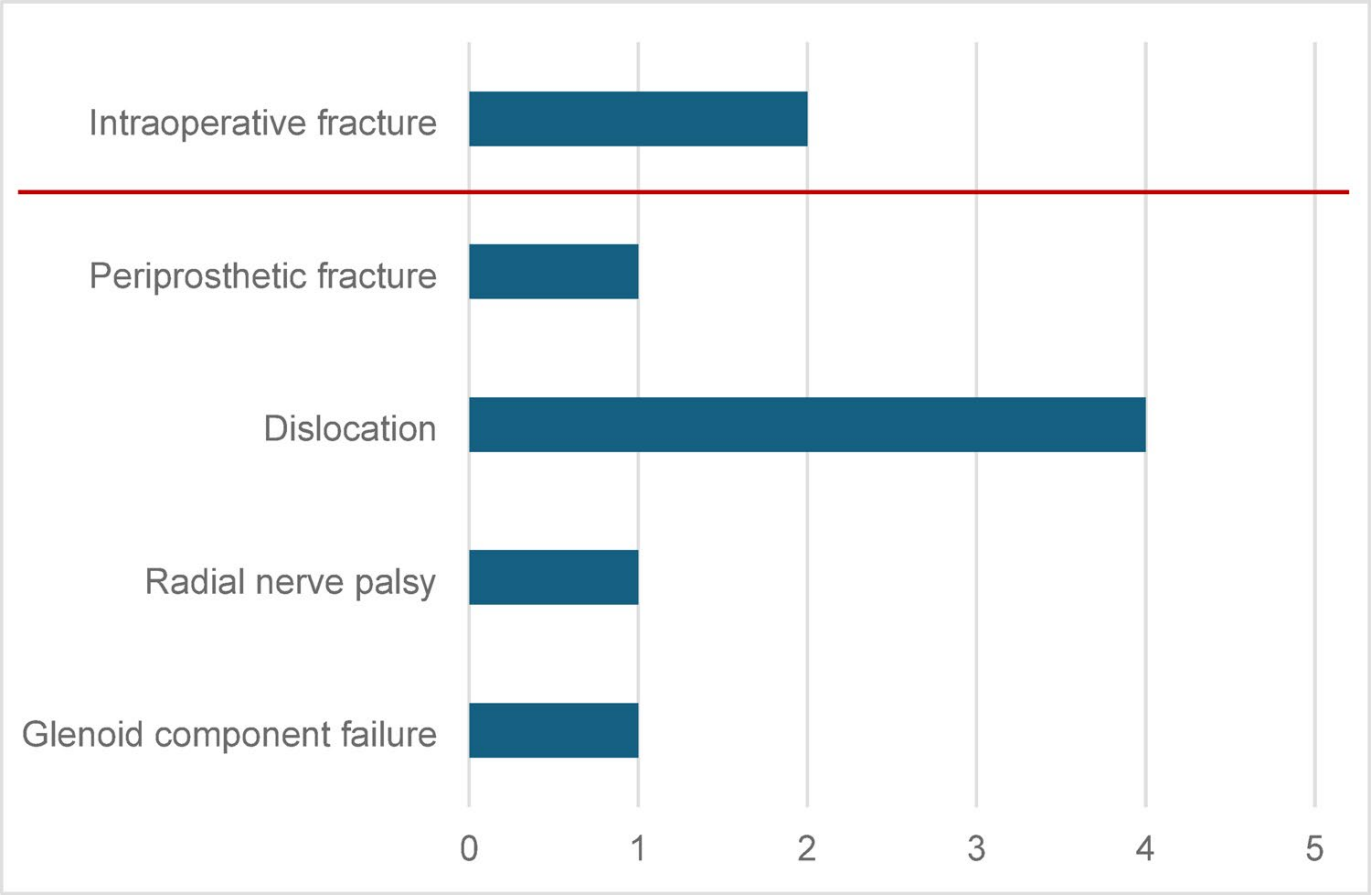
Incidence and types of complications of RSA

During follow-up, 9 complications were observed, corresponding to a cumulative incidence of 7.14%. Two complications occurred intraoperatively, while seven occurred during the postoperative period. Two intraoperative fractures were managed using an orthopedic cable system, and the subsequent postoperative course was comparable to that of other patients (Fig. 4).

Postoperative complications include periprosthetic fracture, dislocation, radial nerve palsy and glenoid component failure. The periprosthetic fracture occurred five years postoperatively following a fall and was treated with plate osteosynthesis. Within the first month after surgery, four dislocations were recorded: two resulted from falls and two occurred during routine movement. In each case, the dislocation was managed by exchanging the inlay for a different size. Additionally, postoperative radial nerve palsy was observed, though it resolved completely after six months. The final complication involved failure of the glenoid component due to aseptic loosening 7.5 years after the initial surgery. Ultimately, due to poor bone quality, the components were extracted, and reimplantation was not performed.

### Radiographic assessment

**Fig. 5:**
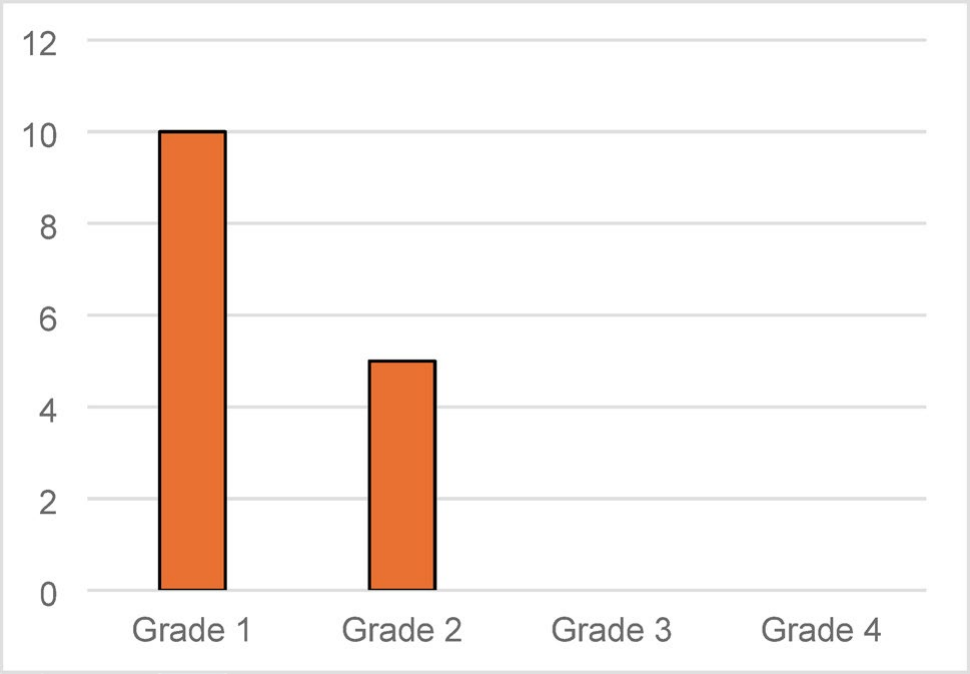
Distribution of scapular notching according to the Nerot–Sirveaux classification (Grades 1–4)

Radiographic complications, defined as pathological findings such as radiolucent lines or loosening, were observed in one patient and led to implant removal. Scapular notching was evaluated separately as a radiographic finding using the Nerot–Sirveaux classification. The most frequent grade was Grade 1 (10 cases), where the defect affected only the inferior pillar of the scapular neck. Grade 2, in which the scapular notch made contact with the inferior screw due to erosion, was observed in 5 cases. Grades 3 and 4 were not observed (Fig. 5).

### Functional outcomes evaluation

Functional outcomes were successfully assessed in 108 patients. The overall mean OSS was 46.34 ± 4.72, reflecting consistent functional performance across the cohort. Scores ranged from 23 to 55, demonstrating variability in individual outcomes. When analyzed by indication, the mean OSS was 45.57 ± 4.81 in the fracture group and 46.98 ± 4.58 in the elective (glenohumeral osteoarthritis) group, with no statistically significant difference between groups (p = 0.094).

### Range of motion (ROM)

The overall range of motion demonstrated satisfactory postoperative shoulder function, with moderate variability across all measured planes. Flexion and abduction reached functional ranges, while extension and adduction remained more limited, as expected following reverse shoulder arthroplasty. External rotation exceeded internal rotation on average. Comparative analysis of range of motion between the glenohumeral osteoarthritis and fracture cohorts revealed no statistically significant differences. The glenohumeral osteoarthritis group showed a tendency toward higher flexion, extension, and internal rotation. External rotation tended to be higher in the fracture group; however, this difference did not reach statistical significance (p = 0.053). Adduction was comparable between groups. Detailed numerical values are presented in Table 2.

**Table 2:** Range of motion of patients after RSA

Active motion	Overall	Glenohumeral osteoarthritis	Fracture	*p* values
Shoulder flexion (mean ± SD)	119.68° ± 25.61°	122.21° ± 24.97°	118.04° ± 26.64°	0.384
Shoulder extension (mean ± SD)	13.54° ± 10.08°	14.76° ± 10.69°	11.47° ± 7.81°	0.311
Shoulder abduction (mean ± SD)	112.16° ± 25.50°	111.22° ± 24.65°	114.26° ± 26.78°	0.543
Shoulder adduction (mean ± SD)	12.29° ± 9.88°	13.16° ± 10.79°	12.14° ± 8.6°	0.729
Shoulder external rotation (mean ± SD)	36.88° ± 8.88°	33.75° ± 8.69°	40.00° ± 7.91°	0.053
Shoulder internal rotation (mean ± SD)	24.78° ± 9.94°	25.45° ± 10.97°	24.17° ± 8.86°	0.559

## Discussion

In this study, we evaluated mid- to long-term implant survival, complications, radiographic findings, and functional outcomes in patients undergoing RSA for advanced glenohumeral osteoarthritis and complex proximal humeral fractures. Overall implant survival was satisfactory, with stabilization of survival rates beyond the early postoperative period. The overall complication rate was low. Due to the limited number of events, no definitive comparison between indications can be made. Although no statistically significant predictors of failure were identified, a trend toward increased risk was observed in patients treated for fracture indications and in those receiving uncemented stems. These findings suggest a potential trend; however, no definitive effect of surgical indication on implant survival can be concluded. When comparing fracture and elective indications, our findings are consistent with large registry-based data, suggesting that surgical indication alone may not be an independent determinant of implant longevity when contemporary implant systems and standardized surgical techniques are used. Functional outcomes were favorable across the cohort, with a mean Oxford Shoulder Score of 46.34 ± 4.72 and satisfactory ranges of motion at final follow-up.

To date, limited data are available regarding the long-term implant survival of RSA. In our cohort, the overall implant survival reached 93.4% and remained stable throughout the follow-up period, indicating durable mid-term performance. However, our analysis revealed that survival rates differed based on the primary diagnosis. While patients with glenohumeral osteoarthritis maintained a high and consistent survival rate of 98.3%, the survival rate among those treated for fractures decreased from 95.1% at one year to 86.4% at eight years. A comparable study evaluating the same prosthesis design reported a 14 years implant survival rate of 95.7%. Although our overall survival rate was slightly lower, it remains within the upper range of published outcomes and supports satisfactory implant durability. Other studies have demonstrated a 10-year implant survival of approximately 94%, while additional reports describe survival rates ranging between 80 and 95.2% at ten years, depending on patient population and study methodology [19–22]. Recent evidence indicates that even in proximal humeral bone loss, advanced reconstructive strategies do not confer lower complication or reoperation rates, supporting our findings that surgical indication alone may not be a dominant determinant of outcomes [23]. When comparing these results to our cohort’s survival rates by diagnosis, the survival rate for glenohumeral osteoarthritis (98.3%) was particularly high, exceeding values reported in the literature. For patients treated for fractures, the long-term survival rate was lower (86.4%), yet it remained within the range reported in comparable studies. The low number of revision events limits the robustness of the Kaplan–Meier survival estimates and multivariable Cox regression analysis, with a low event-to-variable ratio increasing the risk of model overfitting. Therefore, the identified associations should be interpreted with caution and considered exploratory rather than confirmatory.

The overall complication rate of 7.14% observed in our cohort is relatively low compared with previously published rates, which range widely from 1 to 55%. This variability is largely attributable to differences in surgical indication, implant design, follow-up duration, and definitions of complications [24–26]. In a study evaluating the same prosthetic system (Affinis), a comparable complication rate of 7.8% was reported [16]. Notably, no infectious complications occurred in our series. Given that infection is commonly reported as one of the leading complications following RSA, this finding is noteworthy and may reflect careful patient selection, perioperative management, or sample size limitations. The majority of complications in our cohort were trauma-related or were successfully managed without compromising implant retention. The only event directly affecting implant survival was aseptic loosening of the glenoid component. Consequently, definitive implant loss due to revision was rare, which aligns with the high implant survival rates observed at mid-term follow-up.

Functional outcomes following RSA are critical to restoring patient quality of life. Current literature regarding RSA outcomes reports OSS ranging from 34 to 40 [27–29]. While the primary objective of RSA is to achieve functional restoration comparable to healthy baseline levels, our study observed a mean postoperative OSS of 46.34. This figure exceeds previously reported postoperative values; however, in the absence of preoperative functional data, it should be interpreted as final postoperative status rather than evidence of superior recovery. In contrast to our indications, reverse shoulder arthroplasty performed after proximal humeral tumor resection has been associated with inferior functional outcomes due to extensive bone and soft-tissue compromise [30].

At the final follow-up, the range of motion was satisfactory. Published data demonstrate postoperative RSA values ranging from 112 to 140° for flexion (anterior elevation), from 103 to 120° for abduction, and from 9 to 45° for external rotation [19, 21, 31]. The mean ROM observed in our cohort was within established ranges, frequently reaching the upper limits described in the literature. No statistically significant differences in postoperative ROM were found between the glenohumeral osteoarthritis and fracture cohorts. While glenohumeral osteoarthritis patients trended toward higher flexion and internal rotation, the fracture group exhibited slightly better abduction and external rotation. These results suggest that postoperative ROM values were broadly similar between groups; however, the absence of preoperative functional data limits conclusions regarding comparative recovery.

Several limitations should be considered when interpreting our findings. First, this was a single-center study using a single implant system, which enhances internal consistency but may limit the generalizability of the results to other prosthetic designs, fixation philosophies, or surgical techniques. The retrospective design introduces a potential risk of selection and information bias. In addition, baseline differences between fracture and elective groups may have influenced the results despite adjustment using multivariable regression analysis. Residual confounding cannot be fully excluded. Another important limitation is the relatively low number of revision events, which reduces the statistical power of the analysis and increases the risk of Type II error. The non-random allocation of fixation type introduces potential selection bias, as cemented fixation was more frequently used in fracture cases and in patients with poorer bone quality. In addition, although radiographic evaluation was performed using standardized true AP and axial projections, plain radiographs may underestimate subtle radiolucent lines, early loosening, or three-dimensional scapular notching compared with advanced imaging modalities. Finally, functional outcomes were assessed at the latest follow-up without systematic preoperative baseline scores, precluding precise quantification of clinical improvement and limiting comparison of recovery trajectories between fracture and elective indications. The use of a single patient-reported outcome measure OSS may limit the sensitivity of functional assessment, particularly in the presence of a ceiling effect. Although the mean follow-up duration exceeded four years, the minimum follow-up of 12 months and variability across patients may limit the detection of late complications.

## Conclusion

Reverse shoulder arthroplasty demonstrated satisfactory mid- to long-term implant survival and favorable postoperative outcomes in both fracture and elective indications. Although fracture cases showed numerically lower survivorship, no statistically significant independent effect of surgical indication on implant failure was demonstrated in this cohort. The findings should be interpreted with caution given the retrospective design, limited number of revision events, and absence of preoperative functional data.

## Data Availability

No datasets were generated or analysed during the current study.
